# ‘Weighing’ Losses and Gains: Evaluation of the Healthy Lifestyle Modification After Breast Cancer Pilot Program

**DOI:** 10.3389/fpsyg.2022.814671

**Published:** 2022-03-25

**Authors:** Dana Male, Karen Fergus, Shira Yufe

**Affiliations:** ^1^Tom Baker Cancer Centre (TBCC), Department of Psychosocial Oncology, Alberta Health Services, Calgary, AB, Canada; ^2^Psychosocial Oncology Laboratory, Department of Psychology, York University, Toronto, ON, Canada; ^3^Odette Cancer Centre, Sunnybrook Health Sciences Centre, Toronto, ON, Canada

**Keywords:** breast cancer, survivorship, online intervention, group, health behavior change, holistic, psychosocial oncology

## Abstract

**Objectives:**

This pilot study sought to develop and evaluate a novel online group-based intervention (Healthy Lifestyle Modification after Breast Cancer; HLM-ABC) to help breast cancer survivors (BCSs) make healthy lifestyle changes intended to yield not only beneficial physical outcomes (i.e., weight loss, reduced body mass index) but also greater behavioral (e.g., increased physical activity, healthier eating), and psychosocial well-being (e.g., self-efficacy, motivation, body image).

**Methods:**

An exploratory single-arm, mixed-method triangulation design was employed to evaluate the feasibility and preliminary effectiveness of the HLM-ABC intervention for overweight BCSs. Fourteen women participated in the 10-week intervention and completed quantitative measures of the above-mentioned outcomes at baseline, post-treatment, 6-month, and 12-month follow-up time points. Qualitative data were obtained post-treatment via semi-structured interviews and a treatment satisfaction questionnaire.

**Results:**

Participants lost an average of 2.83% of their baseline weight (*M* = 196.65; *SD* = 38.59) by 1-year follow-up (*M* = 191.29; *SD* = 33.91), equal to a small effect size (*d* = –0.37). Despite achieving only modest weight loss, participants achieved meaningful gains in the form of increased physical activity (*d* = 0.2), discovery of gratifying movement, more intuitive eating habits (*d* = 1.12), greater bodily and emotional awareness, and positive shifts in beliefs about being able to make healthy choices regarding food (*d* = 0.63) and physical activity (*d* = 0.38). Furthermore, they demonstrated a slight improvement in body image (*d* = 0.36) and described feeling more self-compassionate, empowered, and acknowledging of variables beyond control (i.e., hormonal therapy, unsatisfactory surgery) that can present barriers to change.

**Conclusion:**

After completing a 10-week online program, participants achieved meaningful and lasting changes on a number of healthful indicators, even when this did not correspond with a significant reduction in weight. Findings highlight the complex, multifaceted nature of “health” and lend support for promotion of healthier lifestyle following cancer treatment that encompasses not only physical weight, but also behavior, psychosocial well-being, and (often unmodifiable) circumstances such as life-preserving hormonal treatments.

## Introduction

The overall 5-year net survival for BC is 87%, varying from 22% for stage IV to nearly 100% for stage I (Canadian Cancer Society [CCS], 2022a). In light of advances in early detection and increased survival rates, there remain numerous long-term challenges for women following BC treatment. Weight gain is one of the most prevalent yet less commonly discussed survivorship concerns, with a reported 50–96% of women who gain weight after diagnosis and treatment of BC (Wang et al., 2022). The most recent data available from a nation-wide American health survey (Greenlee et al., 2016) reported that approximately 30–35% of breast cancer survivors sampled were obese.

Possible explanations for treatment-related weight gain are a combination of type and length of chemotherapy, increased fatigue, reduced physical activity (PA) and resting energy expenditure, increase in energy intake, and development of amenorrhea and/or menopause (Campbell et al., 2007). Excess weight and body fat distribution have been identified as important modifiable risk factors for BC survivors, given their association with other health conditions (e.g., type II diabetes, heart disease, stroke), as well as increased risk for cancer recurrence and mortality (Protani et al., 2010; Ligibel, 2011; Ligibel and Strickler, 2013). In the case of BC specifically, increased fat results in greater production of estrogen, insulin, leptin and pro-inflammatory cytokines, and lesser production of sex hormone binding globulin, all of which have been linked to the promotion of BC and tumor growth (Rock et al., 2013). Being overweight is also associated with a range of psychological difficulties including low self-esteem, mood disorders, eating disorders, chronic pain, sleep disturbances, and reduced quality of life (QoL) (Collins et al., 2016). Psychological issues can underlie the development of unhealthy behaviors and excess weight; they can also occur as a result of ongoing weight struggles.

### Physical Activity and Eating Habits

Along with obesity, lack of PA is considered to be one of the most important health determinants in breast cancer survivors (BCSs) who have completed adjuvant treatment (Sinicrope et al., 2010). BCSs experience severe side effects during and following treatment that can directly limit mobility and engagement in PA (Whitehead and Lavelle, 2009; Schmidt et al., 2017).

Research consistently demonstrates the importance of PA during BC survivorship. The health benefits of PA appear to be true for BCSs regardless of tumor stage, cancer treatment, smoking habits, menopausal status, body composition, and weight and have been demonstrated in large and small sample-sizes alike, across various cultures (Schmid and Leitzmann, 2014). Guidelines of 30 min per day of moderate to vigorous daily activity or 150 min of moderate (or 75 min of vigorous) activity per week have been published by the Canadian (Canadian Cancer Society [CCS], 2022b) and American (American Cancer Society [ACS], 2020). Cancer Societies, respectively. Dietary guidelines for BCSs have also been developed (e.g., intake of fruits of vegetables, limited intake of foods high in fat and sugar, limited alcohol consumption) (Gandini et al., 2000; Rock et al., 2012; Hamer and Warner, 2017) and represent an important modifiable part of a woman’s overall lifestyle that can contribute to healthy weight management.

Despite research demonstrating that diet and exercise interventions can lead to improvements in physical and mental health, there remains a lack of consistent evidence that these changes are maintained over time (Campbell et al., 2012; Greenlee et al., 2012). Studies investigating the benefits of moderate-to-vigorous physical activity (MVPA) following diagnosis of BC reveal that such benefits seem to only be maintained for as long as exercise behaviors continue (Ritvo et al., 2017). Therefore, the maintenance of healthy behaviors over time following active completion of such interventions is paramount. Furthermore, evidence suggests that interventions that account for psychosocial factors and include self-directed professionally guided PA, follow-up behavioral prompts, and at least four sessions of related counseling are associated with successful promotion of PA (Ritvo et al., 2017).

### Self-Concept

BCSs undergo a variety of physical and functional changes that impact their self-image (Male et al., 2016). Thirty-one to 58% of BCSs consider themselves to be less attractive and more dissatisfied with their bodies than they were before treatment (Andrzejczak et al., 2013), 73% report feeling less desired, 44% uncomfortable exposing their body, and 38% less confident (Ussher et al., 2012). Not surprisingly, BCSs score relatively worse on measures of body satisfaction than do women in the general population (Raggio et al., 2014) and, in fact, score similarly to obese women seeking weight-loss treatment (Foster et al., 1997), implying especially low body satisfaction among obese BCSs. The literature suggests that body image concerns amongst BCSs persist over time (Brunet et al., 2013; Raggio et al., 2014; Male et al., 2016; Kang et al., 2018). One study found that women felt similarly about their body image 3 years following mastectomy as they did 10 months after surgery (Fallbjörk et al., 2012). Another study found that 31% of stage II-III breast cancer survivors struggled with body image 4 years after surgery, and 27% continued to struggle 7 years post-surgery (Falk Dahl et al., 2010).

### Theoretical and Applied Underpinnings of Program Development

#### Self-Determination Theory

Self-determination theory (SDT) (Ryan and Deci, 2017) posits that human beings have inherent tendencies to explore, engage, and understand the world around them, and to internalize social norms and rules; these strivings are reliably driven by basic needs of feeling competent, autonomous, and related. SDT in the context of weight loss and healthy eating indicates autonomous motivation predicts greater treatment adherence and weight loss, as well as sustained exercise and weight loss at follow-up (Williams et al., 1996). Conversely, research demonstrates that when people are extrinsically motivated to engage in healthy eating and exercise (i.e., for financial incentive), they enjoy these activities less and have poorer weight loss outcomes (Moller et al., 2014).

#### Mindfulness and Self-Awareness

The concept of mindfulness has been applied to health behavior change in the practice of behavioral self-monitoring. Self-monitoring of diet and PA is a well-established part of effective weight loss interventions, and those who track their eating habits more regularly and completely seem to have the most successful outcomes (Burke et al., 2011). Mindfulness can also be practiced in the form of “mindful” (Kristeller and Lieberstein, 2016; Wnuk and Du, 2017) or “intuitive” eating (Tribole and Resch, 2012). This practice involves non-judgmental awareness of physical and emotional sensations (e.g., hunger and satiety cues) related to eating and intentional choices regarding the same. A systematic review of mindfulness-based interventions for obesity found that 86% of studies demonstrated improvements in eating habits (O’Reilly et al., 2014).

#### Motivational Interviewing

Motivational interviewing (MI) is “a collaborative conversation style for strengthening a person’s own motivation and commitment to change” (Rollnick and Miller, 2013, p. 23). MI places emphasis on individuals’ inherent tendency toward growth, which can be optimized through various psychological processes. MI has been adapted (Clifford and Curtis, 2016) and successfully implemented in health behavior change interventions with patient and non-patient samples (Pudkasam et al., 2018). It has been demonstrated to be a promising approach to promoting PA among BC patients and survivors (e.g., Sheppard et al., 2016).

#### General Systems Theory

General systems theory (GST) (von Bertalanffy, 1950, 1968) provides a framework to understand phenomena from a holistic approach—that is, how distinct parts of a complex, organized system interrelate and operate as a whole toward a broader purpose (Mele et al., 2010). Through a systems lens, a person’s weight or body composition represents only one aspect of their broader health, comprised of a multitude of personal behaviors, emotions, physiological sensations, and thoughts that function together within a larger social context to achieve a state of homeostasis, or what may be referred to as health status. From this view, there are a variety of adaptable ways in which a person (and their environment) can influence the state of their health. In order to achieve sustained change, such as weight loss or other physical health benefits, one must consider how, to what extent, and at what pace, a person’s broader lifestyle and context is likely to accommodate, or reject, such a shift in the system’s homeostasis.

#### National Institute for Health and Care Excellence Guidelines

The National Institute for Health and Care Excellence (NICE) is aimed at improving health and social care by providing evidence-based recommendations, development of standards for health care providers, and provision of information services. The following guidelines were developed by NICE for providers of lifestyle weight management programs based on effective weight loss strategies, and were incorporated into the Healthy Lifestyle Modification After Breast Cancer (HLM-ABC) intervention protocol: incorporate multiple lifestyle components including diet, PA, and other behavior changes; develop by a multidisciplinary team (e.g., dietitian, psychologist); focus on long-term lifestyle change; establish specific and agreed-upon dietary targets tailored to individual needs and goals without “banning” foods or food groups; consider individual input from a registered dietitian; discuss reduction of sedentary behavior and types of PA that can easily be integrated into everyday life and maintained over time (e.g., walking); integrate a number of different behavior-change strategies that involve education, problem solving, goal-setting, social support or changes in one’s environment that can facilitate lifestyle change, self-monitoring, and individualized feedback; tailor to the needs of participants so that the program is accessible and convenient to all; monitor indicators of behavior change and participants’ personal goals throughout the course of the intervention; and adopt a respectful, non-judgmental approach (National Institute for Health and Care Excellence, 2014).

#### Cognitive Behavior Therapy

Cognitive behavior therapy (CBT) formulates problems in living (e.g., obesity, sedentariness) according to a model of how cognitions, emotions, physical sensations, and behaviors reciprocally influence one another (Padesky and Greenberger, 1995) and seeks to improve functioning by modifying thoughts and behaviors. CBT has been demonstrated to be an effective weight loss treatment (Munsch et al., 2007; Cooper et al., 2010; Leal and Ramos, 2012), and particularly with BCSs (Mefferd et al., 2007). The research suggests that the efficacy of CBT for weight management may be increased when delivered in a group format due to the suspected benefits of mutual support, and when combined with individualized nutritional and PA strategies (Leal and Ramos, 2012).

## Materials and Methods

### Study Aims

There is a need to develop holistic health interventions that address the deleterious physical effects of BC treatment as well as the disturbances in self-and body-image arising from these, which are unlikely to resolve naturally. Albeit there are exceptions (e.g., Cohen and Narayanan, 2021), the relative dearth of comprehensive, sustainable health promoting programs for women recovering from BC led to the development of the HLM-ABC intervention (Yufe et al., 2019). The aim of this study was to evaluate the feasibility and preliminary effectiveness of the HLM-ABC program in order to determine the potential justification for further development and larger-scale investigation. From March to July 2017 the program was piloted in an asynchronous online format and employed an exploratory single-arm, mixed-method concurrent triangulation design for the purposes of evaluating the preliminary effectiveness of the HLM-ABC program. Given the small sample size and exploratory nature of this pilot, qualitative and quantitative methods were triangulated in such a way to increase “comprehensiveness” and “confidence” (O’Cathain, 2010, p. 577) in the findings.

#### Research Question

Does this intervention demonstrate promise in terms of helping BCSs achieve and maintain greater physical (weight, body mass index (BMI), and waist circumference), behavioral (intuitive eating and PA levels), and psychosocial (attitudes toward change, psychological distress, QoL, and body image) health?

#### Hypothesis

This program demonstrates feasibility and preliminary effectiveness in terms of improving and maintaining participants’ physical, behavioral, and psychosocial health as indicated by quantitative and qualitative indicators assessed at baseline, post-treatment, 6-months follow-up, and 12-months follow-up.

### Recruitment

Staff oncologists, nurses, dieticians, and physiotherapists at the study site (i.e., cancer center) were informed about the study through internal email and presentations delivered at interprofessional rounds by the primary investigator/first author. These healthcare professionals actively informed patients of the study and obtained their verbal consent to be contacted by a member of the research team for a telephone screening interview. The study was also advertised to patients through flyers posted at the cancer center, as well as through community-based organizations.

**FIGURE 1:**
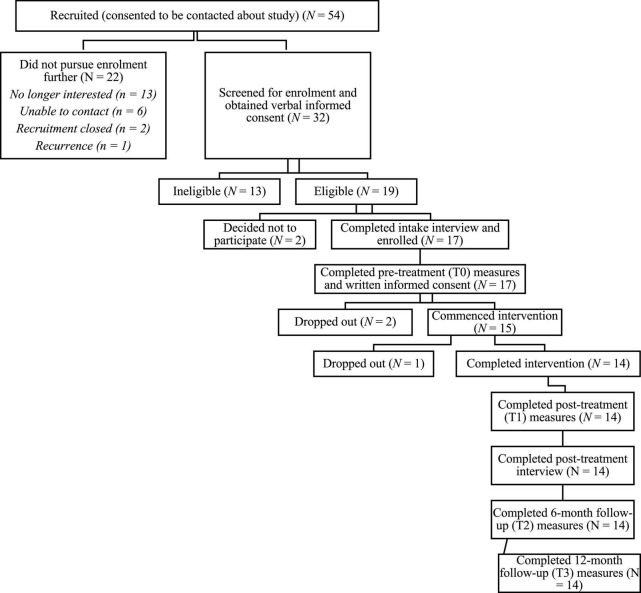
Participant recruitment and retention flowchart.

Prospective participants were contacted by a Research Coordinator (third author, then a clinical psychology graduate trainee) who scheduled a screening interview. During this interview, the Research Coordinator detailed information about the purpose and procedures of the study, obtained informed consent, and determined eligibility based on the following inclusion criteria: (1) be female; (2) be 21 years or older, (3) have been diagnosed with primary BC (stages I-III), (4) completed active treatment within the previous 5 years, (5) have a BMI above 25 (“overweight” category) or report a weight increase of 10 pounds or more post-treatment, (6) be comfortable using, and have access to, a computer and secure Internet connection, and (7) can read and write in English. Exclusion criteria included: (1) current diagnosis of metastatic cancer, (2) diagnosis of a mental health condition that would interfere with their own, or another group members’ ability to benefit from group (e.g., psychosis), (3) diagnosis of an additional unmanaged/untreated medical condition, (4) plans to undergo a medical procedure within the next year, and (5) plans to participate in another structured weight loss program or take weight loss medication within the next year. Figure 1 provides a visual diagram of participant recruitment and retention.

### Procedures

#### Research Ethics Board Approval

Ethics approval was obtained from Sunnybrook Health Sciences Centre (SHSC)’s Research Ethics Office (339-2014) and York University’s Office of Research Ethics (Sunnybrook Approval –339-2014). The trial has also been registered at clinicaltrials.gov, under the ID CBCF-092014.

#### Baseline and Repeated Outcome Measures Data Collection

Participants (*n* = 12) were scheduled by one of the co-investigators (first author) to visit the cancer center to complete baseline data and repeated outcome measures. During the baseline visit (T0), participants reviewed and signed the informed consent form (verbal consent was already obtained during the phone intakes), as well as completed a one-time demographic/medical questionnaire. Repeated measurements were collected at pre-treatment (T0), post-treatment (T1), 6-months follow-up (T2), and 12-months follow-up (T3) and involved measurement of body weight, height, and waist circumference by a registered dietician on site, and completion of a questionnaire package including behavioral and psychosocial self-report measures. Upon completion of the program (T1), in addition to completing the standard repeated measures, participants also completed a one-time Treatment Satisfaction Questionnaire (TSQ).

Two (*n* = 2) of the participants resided rurally and were unable to attend the central cancer center for data collection. These women were emailed password-protected copies of the self-report measures to be completed anonymously and returned electronically via email. These participants solicited the involvement of their primary care provider (i.e., family physician, nurse practitioner) to collect their anthropometric measurements remotely, according to the same written instructions used by the dietitians at the local study site.

#### Post-treatment Interview

Upon completion of the intervention, participants engaged in a post-treatment interview (PTI) conducted over the telephone by a research assistant who was unknown to the participants. The interviews were semi-structured according to an interview protocol (see Supplemental Material), approximately 60 min in duration, audio-recorded, and intended to solicit individualized, open-ended feedback from participants about their experiences in the HLM-ABC intervention.

#### Program Delivery

The HLM-ABC program is a 10-week at-home, group-format lifestyle intervention that was delivered entirely online using “Moodle,*”* an open-source “learning management system.” The platform features interactive, closed-access exchange of information via sharing and uploading of documents, as well as asynchronous communication via discussion forums. Participants were expected to log in, at least once each week but ideally more often, to the secure website using a personal identification and password. They were expected to review and discuss weekly psychoeducational material (i.e., videos) geared at teaching and promoting new and healthier ways of thinking, feeling, and behaving. Discussion forum about the program content and participants’ reactions to it were posted asynchronously on the website’s discussion board. Participants were able to access the group at any time, from any personal computer or device, for the duration of the intervention.

At the beginning of each week, the two co-facilitators (who also occupied roles of co-investigators) alternated posting a message on the discussion forum to introduce that week’s topic, upload the relevant psychoeducational material, and pose questions to facilitate group discussion. They provided clear instructions about the weekly homework and managed the discussion forum daily to respond to questions and promote group interaction. At the end of the week, participants were expected to submit their homework assignments online (which were reviewed by the facilitators who returned individualized feedback), and comment on their experience on the discussion board. Table 1 provides a summary of the educational content and homework assignments delivered over the course of the 10-week intervention.

**TABLE 1:** Healthy lifestyle modification after breast cancer (HLM-ABC) intervention weekly outline.

Week	Topic	Educational Content	Practice Homework
Initial log-in	Welcome/Practice	Introduction to facilitators, group format and responsibilities, general topics to be covered	(1) Practice assignment (2) Introductions
1	Getting Started	Foundational principles and conceptual framework Behaviors, Emotions, Sensations, Thoughts (BEST), balanced “blue zones, systemic balance, 80–20% rule, shifting from external to internal focus, daily planning”	(1) Food List (2) Weekly diary
2	Eating Consciously/Intuitively	“What” and “How” of eating Plate method (proportions, Canada Food Guide recommendations) Snacks as bridge between meals Hunger scale and hunger type	(1) Daily diaries (2) Pause, distract, check-in (3) My danger zones
3	Let’s Get Moving	Tips for increasing and maintaining activity Behavioral activation Steady climb vs. final destination	(1) Diaries (2) My Satisfying Movements (3) Putting my Movements to the Satisfaction Test
4	Barriers to Staying on Course	Planning for inevitability of ‘falling “off track” Triggers and barriers 3 saboteurs (Drill Sergeant, Rebel, Quitter) S.U.R.E. thinking (sudden, unrealistic, rigid, extreme)	(1) Diaries (2) Reviewing the play
5	Overcoming Barriers (Staying on Course)	Cultivating your Inner Coach G.R.A.B. principles (gradual, realistic, adaptable, balanced) Coach’s tips: set schedule, show up prepared, realistic standards, check in often, recognize your efforts	(1) Diaries (2) Call in the Coach
6	Getting Emotionally Aware and Practicing Self-Care	Reasons for eating The dangerous cycle of emotional eating Basic emotion states and triggers Eating as a “Band-Aid” for emotional discomfort/pain Interrupting the emotional eating cycle: Take C.A.R.E. (Catch, Acknowledge, Recognize, Engage) Emotions and corresponding needs and action tendencies Proactive self-care (“oxygen mask” metaphor)	(1) Diaries (2) Taking C.A.R.E. with emotional “falls” (in-the-moment self-care) (3) Investing in Me (proactive self-care)
7	Body Image and Self Esteem	Defining body image How breast cancer relates to body image Respecting/accepting your body The Body Bully vs. Self-Compassion/Coach	(1) Diaries (2) Strengthening your self-compassion reflex (3) Check the checking
8	Reviewing the Journey	Review of major principles and strategies B.E.S.T. “Road Map” B.E.S.T. Players	(1) Review and practice exercises from “Repertoire of B.E.S.T. practices”
9	Looking Ahead	Anticipation of set-backs, relapse prevention, troubleshooting for maintenance Focus on group discussion, consolidation, and support	(1) Repetition and practice of homework for feedback
10	Take-aways and goodbyes	Reflect upon progress and mutual support Consolidate gains Group discussion, consolidation, and support (no video)	(1) Repetition and practice of homework for feedback

### Measures

Preliminary effectiveness was assessed by triangulating qualitative findings from the PTI data with quantitative change scores on a number of standardized outcome measures. These outcome measures, summarized below, assessed a range of anthropometric (weight, waist circumference, BMI), behavioral (intuitive eating, PA habits), and psychosocial (motivation, self-efficacy, QoL, body image). Repeated measures were collected at pre-treatment (T0), post-treatment (T1), 6-months follow-up (T2), and 12-months follow-up (T3).

#### Physical/Anthropometric Health

Weight was measured using a physician’s scale. Waist circumference was measured by aligning the bottom edge of a non-elastic measuring tape with the top of the hip bone and wrapping around the waist. BMI was calculated by dividing participant weight (kg) by their height (m^2^).

#### Behavioral Health

Eating habits were measured using the Intuitive Eating Scale—2 (IES-2; Tylka and Kroon Van Diest, 2013). The IES-2 is a 23-item questionnaire that assesses an individual’s degree of intuitive eating, which has been described as an adaptive way of eating, with strong connections to our internal physiological hunger and satiety cues (Tribole and Resch, 2012). The IES-2 has been found to be internally consistent, with Cronbach’s coefficient alpha of 0.87 for the total 23-item IES-2 and similarly high for the subscales (Tylka and Kroon Van Diest, 2013) and total and subscale scores have demonstrated good test-retest reliability (Tylka and Kroon Van Diest, 2013). Cronbach’s alpha for the total IES-2 has been found to be very reliable according to a validation study with a sample of 762 BCSs who were overweight or obese (Cronbach’s alpha of 0.93 for entire IES-2 (Nejati et al., 2020).

Physical activity was measured using the Godin Leisure-Time Exercise Questionnaire (GLTEQ; Godin, 2011). The GLTEQ is a self-report measure consisting of three questions about the frequency and duration of mild, moderate, and strenuous exercise that one typically engages in during a week. The GTLEQ is a commonly used and validated questionnaire among cancer survivors. It has demonstrated adequate construct validity (ranging from *r* = 0.32 to 0.45 when compared to accelerometry) and reliability (intraclass correlation coefficient of 0.74 over a two-week interval) (van Poppel et al., 2010).

#### Psychosocial Health

Psychosocial well-being was assessed according to measures of motivation/attitudes toward change and emotional well-being. The Change Questionnaire (CQ; Miller and Johnson, 2008) asks respondents to identify what they are considering changing (e.g., living a healthier lifestyle) and responding to 12-items about their intentions and motivation to make this particular change. The CQ has demonstrated good internal consistency and test–retest reliability (Miller and Johnson, 2008).

The Exercise Self-Efficacy Scale (E-SES) is a 5-item questionnaire measuring one’s personal agency and optimistic self-beliefs about being capable of adopting healthy exercise behaviors. The scale has an internal consistency (Cronbach’s alpha) of 0.88 and has been found to have satisfactory construct validity, as it is significantly correlated with healthy exercise intentions (*r* = 0.33) and behaviors (*r* = 0.39) (Schwarzer and Renner, 2009).

The Nutrition Self-Efficacy Scale (N-SES) is a 5-item questionnaire measuring one’s perception of their own personal agency or control over eating. This scale has an internal consistency (Cronbach’s alpha) of 0.87 and test-retest correlation of *r* = 0.59. The scale has been found to have satisfactory construct validity, as it is significantly correlated with healthy eating intentions (*r* = 0.22) and behaviors (*r* = 0.34) (Schwarzer and Renner, 2009).

The Functional Assessment of Cancer Therapy – For Patients with Breast Cancer (FACT-B; Brady et al., 1997) is designed to measure QoL in BC patients across various dimensions of physical, social, functional and emotional well-being as well as additional breast-cancer specific concerns. Internal consistency is high (α = 0.90, *N* = 295), and subscale alpha coefficients range from 0.63 to 0.86. The scale has also demonstrated test–retest reliability, convergent validity, divergent validity, and known-groups validity (Brady et al., 1997).

Body image was measured using the Body Image Scale (BIS; Hopwood et al., 2001). The BIS was designed to measure body image concerns that cancer patients/survivors may have experienced during the past week, as experienced in the form of negative thoughts, feelings, or behaviors. The total scale and individual items have demonstrated high reliability when tested with BC samples, with Cronbach’s alpha coefficients equal to 0.93, and ranging between 0.92 and 0.93, respectively (Hopwood et al., 2001).

### Analysis

#### Qualitative Analysis

This study employed a thematic analysis (Graneheim and Lundman, 2004; Braun and Clarke, 2006) of the qualitative PTI data. Firstly, audio recordings of the PTIs were transcribed verbatim and uploaded into NVivo 12 software. Each transcript was read through several times by the first author (DM) to develop an overall sense of each participant’s experience in the program. Subsequently, the text was organized into meaning units (segments of text that together represent a distinct thought, idea, or meaning), which were then assigned a “code.” All codes were compared against one another for similarities and differences in meaning, and organized according to higher-order categories. At this stage, codes and categories were analyzed inductively, in that they very closely reflected the manifest data (Braun and Clarke, 2006). Subsequently, categories were examined against one another and as a whole, and through a process of deductively applying the broader research questions, were grouped into higher-order themes. The themes and organizational structure were subjected to a second independent audit by the second author (KF) and a subsequent dialogue and consensus building where differences existed to ensure validity and reliability of findings.

The researchers in this study were themselves instrumental in delivery of the intervention and analysis of the data. As such, it is important to provide the following background information. DM was a Doctoral Candidate in Clinical Psychology and was familiar with the processes and facilitation of online support groups through her master’s thesis research, for which she conducted an in-depth qualitative analysis of archived transcripts of online group discussion (Male et al., 2015, 2017). She also possessed previous clinical experience facilitating several in-person therapy and psycho-educational groups. KF was DM’s academic supervisor as well as a licensed Clinical Psychologist working as a Scientist-Practitioner at SHSC. KF has extensive (i.e., 20 years) research and clinical experience with online and in-person group facilitation in the area of psychosocial oncology.

The epistemological paradigm of this mixed-method study is a combination of both postpositivism-realism and constructivism-interpretivism (Ponterotto, 2005). The quantitative element of investigation acknowledges that the researchers and participants interact and have an influence on one another (especially because the PI/author occupied additional roles of clinician and research coordinator); however, this approach emphasizes independent and objective measurement of the phenomena under quantitative inquiry (e.g., the impact of the HLM-ABC intervention on overweight/obese BCSs’ health, according to various quantifiable indices).

By combining qualitative and quantitative methodology, this study also integrates a constructivist-interpretivist lens. Therefore, this paradigm also assumes that the phenomena being investigated are determined by multiple, different and equally valid experiences of those individuals who participated in the study. Additionally, this approach does not presume that the researchers and participants are independent of one another; it is believed that “truth” is best discovered through joint pursuit and creation, by means of the researcher interacting with participants and interpreting the multiple meanings of reality they bring forth (Ponterotto, 2005).

#### Quantitative Analyses

In order to investigate whether the HLM-ABC intervention was effective at helping breast cancer survivors achieve (and maintain) healthy changes, sample means and standard deviations of the various outcome measures were calculated for each of the four time points. Given this study’s small sample size and the intention of estimating power and sample size for a possible later-phase trial, the main statistic of interest reported is that of effect size (Cohen’s *d*). The magnitude of effect sizes was interpreted according to Cohen’s thresholds of *d* < 0.2 defined as negligible, *d* < 0.5 defined as small, *d* < 0.8 defined as medium, and *d* ≥ 0.8 defined as large.

Pearson’s chi-squared comparisons were conducted to explore the relationships between weight change (classified as less than 5% change in body weight, 5% greater reduction in body weight, or 5% or greater increase in body weight between baseline and 12-months follow-up), and a number of independent variables, including change in motivation (no change, increase; or decrease from baseline to 12-months follow-up). In addition, Fisher’s exact test was conducted to identify the exact probability that the chi-squared statistics were accurate, given that the data violates the chi-square assumption that the expected frequencies in each cell not be below five (significance level set at *p* < 0.05). All statistical *analyses* were conducted using IBM SPSS Statistics 26 software.

#### Mixed-Method Triangulation

A mixed-methods concurrent triangulation design (Gonzalez Castro et al., 2011) was employed to evaluate changes in domains of physical, behavioral, and psychosocial health. Quantitative and qualitative data were collected and analyzed, separately, with a goal of obtaining multiple viewpoints of the phenomena under study (feasibility and preliminary-effectiveness of the program). Subsequently, the independent yet complementary results of these two data sets were merged at the phase of interpretation and discussion to create a cohesive and comprehensive conclusion to the research questions. A mixed-method triangulation approach was deemed especially appropriate for this pilot study given its small sample size and exploratory nature; this method illuminated convergence amongst findings and therefore increased confidence in such, while also revealing variability or exceptions amidst the multiple methods that could not be captured using a quantitative or qualitative method alone. Triangulation of mixed methods has also been established as especially suitable for social sciences and health research (O’Cathain, 2010). In the present study, preliminary effectiveness was assessed by interpreting the quantitative results (i.e., outcome change scores) in light of the converging and/or diverging thematic findings from the qualitative PTI data. The results presented herein represent those findings that are most relevant to the focus of this paper and are part of a broader dataset that is presented in full elsewhere (Male, 2020).

## Results

### Demographics

Demographic information is summarized in Table 2. This pilot study sample consisted of fourteen (*N* = 14) women, with an average age of 52.07 years (*SD* = 9.75, range 29–71 years). The average age at time of diagnosis was 49.21 years (*SD* = 10.56, range 24–70 years), and the average amount of time that had lapsed since the last active treatment (e.g., surgery, chemotherapy, or radiation) was 26.57 months (*SD* = 20.21 months, range 7–64 months). The majority of women (*n* = 9) were married, three were single, and two identified as being in a common-law partnership (one heterosexual and one same-sex relationship).

**TABLE 2:** Demographic characteristics (*N* = 14).

Variable	*n*	%
Ethnicity White/Caucasian Eastern European Italian South African Jewish	11 1 1 1	78.57 7.14 7.14 7.14
Education High school College University	1 4 9	7.14 28.57 64.29
Employment status – missing data (*n* = 12)		
Working	11	91.67
Retired	1	8.33
Annual income – missing data (*n* = 13) $0-$9,999 $25,000-$49,000	2 2	15.38 15.38
$50,000-$74,000	3	23.08
$75,000-$99,000	2	15.38
$100,000+	4	30.77
Number of children None 1	3 2	21.42 14.29
2	7	50.00
3	2	14.29
Stage of cancer I II III	5 6 3	35.71 42.86 21.43
Surgery		
Single lumpectomy	6	42.86
Single lumpectomy followed by	1	7.14
revision to clean margins		
More than one lumpectomy	1	7.14
Unilateral mastectomy (followed by reconstruction)	5 (2*)	21.43 (14.30*)
Single lumpectomy followed by bilateral mastectomy followed by reconstruction	1	7.14
Other treatment		
Chemotherapy	9	64.29
Radiation	13	92.86
Hormonal Therapy	12	85.71
Herceptin Therapy	5	35.7

n, Refers to the number of participants who endorsed each category. *, Refers to a subset of participants from a category.

### Preliminary Effectiveness

This study sought to determine whether the HLM-ABC intervention demonstrates promise in terms of helping BCSs achieve and maintain greater physical, behavioral, and psychosocial health. This was assessed by comparing quantitative measures of such at baseline to those repeated at post-treatment, 6-month follow-up, and 12-month follow-up, and triangulating these results with participants’ qualitative reports of perceived change (Table 3).

**TABLE 3:** Qualitative themes and sub-themes regarding preliminary effectiveness outcomes.

Category	*n*	*%*
**Theme 1: Behavioral Health Changes**		
(1.1) Eating habits	11	79
How they were eating (i.e., mindfully)	10	71
What they were eating	4	29
When they were eating	6	43
More planful	5	36
Eating when hungry (as opposed to emotional)	3	21
(1.2) Moving habits	8	57
Increasing overall amount of movement	6	43
Engaging in more enjoyable and diverse forms of movement	5	36
Approaching movement more flexibly	5	36
Increased importance placed on movement and its inherent benefits	3	21
Increasing movement in little informal ways that add up	3	21
Creating greater accountability through structure	3	21
**Theme 2: Psychosocial Changes**		
(2.1) Mentality/attitudes toward health behavior change		
Adopting a more gradual and continuous approach to change	10	71
Feeling efficacious	11	79
Efforts sustainable over time	9	64
Empowered through increased self-awareness	9	64
More flexible and self-compassionate	9	64
Greater value and prioritization of self-care and nurturance	5	36
(2.2) Self-concept		
Some degree of change	5	36
Unchanged	1	7

*n Refers to the number of participants who endorsed each category. % Refer to the percentage of participants from the overall sample who endorsed each category.*

#### Physical Health

The preliminary effectiveness of the HLM-ABC intervention program on physical health was assessed by measuring weight, BMI, and waist circumference. Pairwise comparisons were performed for each of the outcome measures at T0 to T1, T2, and T3, to determine preliminary effectiveness of the intervention. Effect sizes for each of these values are presented in Table 4.

**TABLE 4:** Pairwise comparisons of anthropometric, behavioral, and psychosocial outcome scores at baseline (T0) with post-treatment (T1), 6-month follow-up (T2), and 12-month follow- up (T3).

Health domain	Variable	T0-T1 *d* [95% CI]	T0-T2 *d* [95% CI]	T0-T3 *d* [95% CI]
Anthropometric	Weight	0.30 + [–0.48, 1.09]	–0.007 [–0.82, 0.80]	0.37 + [–0.42, 1.15]
	BMI	0.32 + [–0.46, 1.10]	–0.019 [–0.83, 0.79]	0.37 + [–0.41, 1.16]
	Waist circumference	0.39 + [–0.43, 1.21]	–0.052 [–0.86, 0.76]	–0.073 [–0.85, 0.70]
Behavioral	Total IES	–0.91+++[–1.73, –0.097]	–0.81+++[–1.65, 0.037]	–1.12+++[–1.96, –0.29]
	GLTEQ	–0.72++[–1.52, 0.08]	–0.27+[–1.046, 0.51]	–0.21+[–1.02, 0.61]
Psychosocial	MCQ	–0.18 [–0.96, 0.60]	–0.23+[–1.04, 0.59]	–0.42+[–1.21, 0.36]
	N-SES	–0.18 [–0.96, 0.60]	0.072 [–0.74, 0.88]	–0.63++[–1.43, 0.16]
	E–SES	0.23+[–0.55, 1.0050]	0.63++[–0.21, 1.46]	0.38+[–0.41, 1.16]
	FACT-B	–0.01 [–0.79, 0.77]	0.36+[–0.46, 1.17]	–0.06 [–0.83, 0.72]
	BIS	0.38+[–0.40, 1.16]	0.11 [–0.74, 0.95]	0.36+[–0.43, 1.14]

*d, Cohen’s d; CI, 95% confidence interval; +, small effect; ++, medium effect; +++, large effect; IES, intuitive eating scale; GLTEQ, Godin Leisure-Time Exercise Questionnaire; MCQ, Motivational Change Questionnaire; N-SES, Nutritional Self-Efficacy; E-SES, Exercise Self-Efficacy; FACT-B, Functional Assessment of Cancer Therapy – for Patients with Breast Cancer; BIS, Body Image Scale.*

##### Weight

Weight (lbs) decreased from pre-intervention (T0) (*M* = 196.65; *SD* = 38.59; range = 159.83–300.49) to post-intervention (T1) (*M* = 194.50; *SD* = 35.24; range = 159.39–279.50). This represents a 1.09% reduction in baseline weight and a small effect. Weight did not change from pre-intervention to 6-months follow-up (T2) (*M* = 193.63; *SD* = 33.29; range = 157.85-264.55). Weight decreased from pre-intervention to 12-months follow-up (T3) (*M* = 191.29; *SD* = 33.91; range = 156.31–260.15), which is a 2.73% reduction in baseline weight and a small effect.

In order to explore individual variability within the sample and determine whether this mean trend in weight change was representative of individuals’ change trajectories, descriptive statistics were examined for each individual participant across all four time points. Using the recommended guideline in the literature of at least 5% reduction in weight from baseline as the cut-off for clinically meaningfully weight loss (Swift et al., 2016), this analysis revealed that five participants in this study lost a clinically significant amount of weight at 12-months follow-up. Weight loss amongst these participants varied from 9.7 pounds (5.33% reduction in baseline weight) to 40.34 pounds (13.42% reduction in baseline weight) between T1 and T4.

##### Weight, Body Mass Index

BMI decreased minimally from pre-intervention (T0) (*M* = 33.51; *SD* = 5.32; range = 27.82–46.84) to post-intervention (T1) (*M* = 33.14; *SD* = 4.75; range = 27.74–43.57). BMI did not change from pre-intervention to 6-months follow-up (T2) (*M* = 33.11; *SD* = 4.59; range = 27.54–41.24). BMI decreased from pre-intervention to 12-months follow-up (T3) (*M* = 32.62; *SD* = 4.71; range = 26.65–40.55), with five participants moving down a BMI category (four went from “class I obese” to “overweight” and one went from “class II obese” to “class I obese”), one participant went up a BMI category from “overweight” to “class I obese,” and the eight others remained in the same BMI category (two remained “overweight,” two remained “class I obese,” three remained “class II obese,” and one remained “class III obese”).

##### Waist Circumference

Waist circumference (cm) increased from pre-intervention (T0) (*M* = 105.15; *SD* = 11.58; range = 88.00–124.00) to post-intervention (T1) (*M* = 105.31; *SD* = 11.61; range = 86.00–124.00), with a small effect. There was essentially no difference in waist circumference from pre-intervention to 6-months follow-up (T2) (*M* = 104.18; *SD* = 12.47; range = 84.50–124.46) or from pre-intervention to 12-months follow-up (T3) (*M* = 105.58; *SD* = 11.06; range = 91.44–124.46).

#### Behavioral Health

Pairwise comparisons of intuitive eating scores and PA scores were calculated at T0 to T1, T2, and T3, and effect sizes are presented in Table 4. These quantitative results were triangulated with qualitative descriptions of altered patterns of eating and moving (Table 3, items 1.1 and 1.2).

##### Eating Habits

Intuitive eating (according to the total IES score), increased from pre-intervention (T0) (*M* = 2.90; *SD* = 0.61; range = 1.91–4.09) to post-intervention (T1) (*M* = 3.25; *SD* = 0.52; range = 2.26–4.22). Intuitive eating also increased from pre-intervention to 6-months follow-up (T2) (*M* = 3.28; *SD* = 0.64; range = 2.43–4.52) and from pre-intervention to 12-months follow-up (T3) (*M* = 3.25; *SD* = 0.56; range = 2.39–4.30). There were large effect sizes for each of these pairwise comparisons.

Participants (*n* = 11; 79%) described a number of *changes made to their eating habits* that they ascribed to the HLM-ABC program (Table 3, item 1.1). These changes corresponded with modifications in (1) *how they were eating* (i.e., more mindfully) (*n* = 10; 71%), (2) *what they were eating* (*n* = 4; 29%), and (3) *when they were eating* (*n* = 5; 36%). With respect to the first of these changes, some of the women described being more thoughtful about their reasons for eating (*n* = 6; 43%). One participant (P14) explained:

…we had to rate the type of hunger we were having— is it bored hunger, is it biological hunger, is it emotional hunger? Like that kind of stuff. I found having to actually stop and keep track of that kind of thing really helped in terms of thinking about why I’m eating, what I’m eating, when I’m eating. And I find, even though I don’t submit that to anybody anymore, I find it still comes to mind when I’m making choices about what I’m eating…

A number of women (*n* = 3; 21%) also spoke about how they developed greater consciousness of their internal bodily sensations around eating and relatedly, their unique eating preferences, which led to more intentional choices. One woman (P04) stated:

…It was actually the first time, I mean it seems obvious that you shouldn’t eat when you’re not hungry…I heard that [laughing], but it didn’t really ever actually sink in. And in this program, I started to realize I ate dinner because dinner was ready. Because everyone else was hungry. And that I don’t overeat massively, but I eat more than I need to. And, you know, instead of feeling just full, sometimes I’m a little too full…I’m thinking about what I’m eating and how I feel [now]… I’ve never ever focused on how food made me feel. Seems so crazy.

In terms of reported changes in *what they were eating*, participants described eating a better balance or proportion of ‘healthy’ foods to ‘unhealthy’ foods (*n* = 3; 21%). They spoke of having changed their eating habits in terms of *when they ate* (*n* = 6; 43%); for example, being more planful to reduce feelings of extreme hunger (*n* = 5; 36%).

##### Moving Habits

The average level of PA across the sample (as assessed using the GLTEQ) increased from “moderately active” at pre-intervention (T0) (*M* = 21.57; *SD* = 19.05; range = 0.00–74.00) to “active” at post-intervention (T1) (*M* = 33.36; *SD* = 18.26; range = 6.00–64.00). This was a medium effect. The level of PA remained essentially the same from pre-intervention to 6-months follow-up (T2) (*M* = 21.38; *SD* = 10.98; range = 6.00–44.00), and increased from “moderately active” at pre-intervention to “active” at 12-months follow-up (T3) (*M* = 25.92; *SD* = 17.10; range = 3.00–60.00). This was a small effect.

Participants (*n* = 8; 57%) described a number of *changes made to patterns of physical activity* (Table 3, item 1.2). Five participants described *approaching movement more flexibly*, adjusting their expectations to be more realistic and thus achievable. For instance, one woman (P02) explained:

I think this is just a more realistic approach to a life change—like a lifestyle change. But I think that, just thinking of it that way- that any kind of movement is valuable, that really helped me because I used to put a lot of pressure on myself that I wasn’t going to the gym for an hour. If I wasn’t doing this much cardio or that much strength training, then it really wasn’t worth it. And so that’s kind of shifted in me; I’m a little more laid back about what I do and how long, as long as I’m doing something.

The other way participants (*n* = 3; 21%) seemed to change their way of thinking about PA was in terms of the *increased importance placed on movement and its inherent benefits*, as opposed to it being a means toward the end of weight loss:

Okay, so in my mind before was that exercise isn’t really going to take weight off, it’s the eating. But now, they’ve changed it to *moving*, which I like way better than *exercise*. And so even though I still feel moving isn’t going to take your weight off, moving is going to give you health (P06).

Several participants also described *increasing their overall amount of movement* (*n* = 6; 43%) and *engaging in more enjoyable and diverse forms of movement* (*n* = 5; 36%). A number of participants (*n* = 3; 21%) described changing the way they move in terms of *increasing movement in little informal ways that add up* over time, such as “making a choice to park my car further away when I go into a store” (P14). Three women described *creating greater accountability through structure*.

#### Psychosocial Health

The HLM-ABC program’s potential to improve participants’ psychosocial functioning was evaluated by triangulating quantitative and qualitative measures of motivation and attitudes toward change, and psychological well-being. Motivation and attitudes toward change was assessed according to scores on the MCQ, N-SES, E-SES, as well as qualitative feedback that emerged from the PTIs. Emotional well-being was assessed based on QoL (FACT-B), body image distress (BIS), and relevant qualitative findings. Pairwise comparisons were performed for each of the repeated outcomes at T0 to T1, T2, and T3. Effect sizes are presented in Table 4. Qualitative themes are summarized in Table 3 (items 2.1 and 2.2).

##### Motivation and Attitudes Toward Change

Motivation for change did not change from pre-intervention (T0) (*M* = 8.92; *SD* = 0.52; range = 7.92–9.75) to post-intervention (T1) (*M* = 9.21; *SD* = 1.44; range = 4.75–10.00). Motivation increased from pre-intervention to 6-months follow-up (T2) (*M* = 9.15; *SD* = 0.87; range = 7.42–10.00) and from pre-intervention to 12-months follow-up (T3) (*M* = 9.40; *SD* = 0.83; range = 7.33–10.00). These effects were small.

According to the Pearson’s chi-squared comparisons analysis, a significant relationship was found between weight and change in motivation, with Fisher’s exact test = 7.94, *p* < 0.05. Participants who experienced a decrease in motivation over the course of the intervention were more likely to have gained weight, those who did not experience a shift in motivation were more likely to also not have experienced a significant change (defined as 5% or more) in weight, and those who experienced an increase in motivation were more likely to have either lost an insignificant (less than 5%) or significant (5% or greater) amount of weight, but not to have gained weight.

Participants’ self-efficacy regarding nutrition (N-SES) essentially remained the same from pre-intervention (T0) (*M* = 14.14; *SD* = 2.69; range = 10.00–20.00) to post-intervention (T1) (*M* = 15.00; *SD* = 3.94; range = 7.00–20.00) and from pre-intervention to 6-months follow-up (T2) (*M* = 14.23; *SD* = 3.61; range = 9.00–20.00). N-SES increased, however, from pre-intervention to 12-months follow-up (T3) (*M* = 15.79; *SD* = 2.83; range = 10.00–20.00). This was a medium effect suggestive that between the time prior to participating in the HLM-ABC and 12-months following completion of this program, participants’ perception of their own personal agency or control over their eating increased.

Participants’ self-efficacy regarding exercise (E-SES) decreased from pre-intervention (T0) (*M* = 12.93; *SD* = 3.27; range = 8.00–20.00) to post-intervention (T1) (*M* = 12.21; *SD* = 4.92; range = 5.00–20.00). E-SES decreased from pre-intervention to 6-months follow-up (T2) (*M* = 11.39; *SD* = 4.79; range = 5.00–20.00). E-SES decreased from pre-intervention to 12-months follow-up (T3) (*M* = 11.79; *SD* = 4.08; range = 6.00–20.00). These effects were small, medium, and small, respectively. Findings suggest that between the time prior to participating in the HLM-ABC program and 6- and 12-months following completion, participants’ perception of their personal agency or control over their exercise habits decreased slightly.

Participants described a number of specific *changes in their mentality or attitudes toward health behavior change* during the PTIs (Table 3, item 2.1). The first of these changes was *adopting a more gradual and continuous approach to change* (as opposed to sudden) (*n* = 10; 71%), as illustrated by the following quote:

I wanted to lose some weight, and, get some exercise, get back on track. And you know…I thought it would come off faster. But that’s okay too. That’s okay. It’s, you know, if I can do a few more pounds in the next 10 weeks, if I just sort of look at it that way… I think we all said that in the first place though, you know, if you look over a year, if you can lose, you know seven or eight pounds in 10 weeks and you stay with it, maybe you lose 20 over a year. And then in 3 years you would have lost 60 pounds.

Several (*n* = 9; 64%) participants spoke about how the program helped them develop a *more flexible and self-compassionate* (as opposed to rigid, extreme and critical) attitude toward accomplishing and maintaining their health goals. Another theme that emerged through the PTIs regarding motivation and attitude toward change was that of *feeling efficacious* (*n* = 11; 79%). This finding seemed to be related to participants’ belief that their efforts to live healthier would be *sustainable over time* (*n* = 9; 64%). Another sub-theme that emerged was that of feeling *empowered through increased self-awareness* (*n* = 9; 64%). One participant (P07) described how the program helped increase her awareness of her internal dialogue and how doing so positioned her to feel more confident in making healthier choices:

I definitely [feel that I am living a healthier lifestyle] … when I go out and it’s like “oh, you know what, I deserve a treat,” I kind of double think. I have had… a bad day where I did buy a bag of potato chips, but it was okay. “Now you’ve done it, you’ve got it over with. Let’s move on, let’s get back.” Whereas before, I’d be all, “You know, who cares? I’m going to be this way, and screw it anyway.” So yeah, it’s helped… I kind of feel more it is in my hands to deal with. Whereas before I would just not be happy and it got to the point where it was like “Okay, I’m just going to keep eating because… I don’t care because I don’t know what the hell to do.“… I feel good that it’s in my hands, I know there is a solution, and… I know what the block is.

Finally, participants (*n* = 5; 36%) spoke of the program shifting their attitude toward health behavior change was in the way it promoted *placing greater value and prioritization on self-care*.

##### Psychological Well-Being

Quality of life, as measured by the FACT-B, remained essentially the same from pre-intervention (T0) (*M* = 104.99; *SD* = 16.36; range = 77.00–128.00) to post-intervention (T1) (*M* = 105.10; *SD* = 18.22; range = 64.00–131.00). QoL decreased from pre-intervention to 6-months follow-up (T2) (*M* = 101.14; *SD* = 21.22; range = 63.00–139.00), and was essentially the same at pre-intervention as 12-months follow-up (T3) (*M* = 105.88; *SD* = 21.71; range = 61.00–133.00). These results reveal that QoL remained generally unchanged, except for a temporary decrease 6-months after treatment, before returning to baseline level.

Body image distress was measured using the BIS, where a lower score is indicative of better body image (i.e., less distress). On average, BIS decreased from pre-intervention (T0) (*M* = 14.48.99; *SD* = 6.66; range = 2.00–25.00) to post-intervention (T1) (*M* = 13.07; *SD* = 7.54; range = 3.00–26.00). This effect was small. Body image distress remained essentially the same from pre-intervention to 6-months follow-up (T2) (*M* = 13.50; *SD* = 7.81; range = 1.00–26.00). Body image distress decreased from pre-intervention to 12-months follow-up (T3) (*M* = 13.14; *SD* = 7.13; range = 3.00–26.00). This effect was small, and suggests that on average, women in this sample were slightly less distressed about their bodies immediately after participating in the HLM-ABC program, and still 1 year following their participation.

Six of the participants spoke about their relationship with their body, which revealed two broad themes regarding self-concept: *some degree of change* (*n* = 5; 36%), *and unchanged* (*n* = 1; 7%). Only one of the participants (P02) described an unchanged, entirely negative experience of her body:

I kind of found that I wasn’t too open to [discussing body image]. I kind of was, I don’t know, I think I have a bit of a barrier… I mean it’s been a while since I’ve gone through reconstruction and I’m just not happy with, you know the way that I look and I don’t know if that’s ever going to change. So, it’s just a topic that makes me feel really angry and kind of bitter… So that’s a big part of my frustration, you know? Like I shouldn’t be 159 pounds.

The other five women described at least some element of their self-concept as having shifted, or being in the process of shifting. Two women described feeling better about their bodies in direct relation to perceiving that they had achieved some tangible physical change (i.e., weight loss, desired change in body shape, size, and/or strength). For example:

Let’s just say I feel better in terms of feeling stronger. I feel like I have more energy, I feel like my mobility is better, I just feel a bit more normal in terms of that aspect instead of before, which was really bad. But because of that, I would say I feel leaner. I don’t actually know if I’ve lost weight… I think may have lost inches maybe but I feel better than when I started. (P19)

The other three women appeared to have “reframed” their experience of their body, regardless of having achieved a desirable amount of weight or feeling as though they directly improved their body image. One woman (P07) described that while she still felt “not good about [her body],” she also felt more “empowered” in terms of recognizing her barriers to weight loss (e.g., medications, unhelpful extreme thinking) and that “finally after all these years, it’s in perspective. It’s not going to be a quick hit.”

## Discussion

Participants in the HLM-ABC program evidenced trends in achievement and maintenance of healthier lifestyle habits up to 1 year following completion, based on a number of physical (i.e., modest but sustained weight loss, downward trend in BMI), behavioral (i.e., higher levels of PA and intuitive/mindful eating), and psychosocial (i.e., slight yet sustained improvements in motivation, self-efficacy regarding eating habits, and body image) outcomes.

### Physical Health

On average, participants in the current sample lost 2.15 pounds between pre-treatment and the time that they completed the HLM-ABC program, approximately 10 weeks later. At 1-year follow-up, the sample weighed an average of 5.36 pounds less than at pre-treatment, meaning that, on average, participants maintained a 2.73% reduction in baseline weight up to 12 months after completing the program.

A minimum of five percent reduction in body weight has been associated with significant health benefits and has been suggested as the cut-off for clinically meaningful weight loss (Rock et al., 2012; Swift et al., 2016; Borek et al., 2018). According to this guideline, five participants (36%) in the current sample maintained a clinically significant amount of weight loss at 1-year follow-up, with one participant having maintained a 40-pound (13.42%) weight reduction. At 12-months follow-up, seven (50%) participants’ weight had not changed significantly (less than 5% reduction) and two (14%) had gained a clinically significant amount weight. In comparison, Christian et al. (2010) found in their review of “low intensity” lifestyle modification interventions that at 12-months follow-up, 29% of participants maintained a weight loss of 5% or greater, 27% lost less than 5% weight, and 43.6% lost no weight or gained weight. A large meta-analysis of 24 randomized controlled trials (*N* = 6,042) of group-based diet and/or exercise interventions for overweight/obese men and women reported an average reduction of 7.58 pounds at 12 months follow-up, varying from 0 to 21.16 pounds. Percent reduction in baseline weight for 23 of the studies revealed an average of 4.83% weight loss. Only 14 of these interventions yielded a minimum of 5% weight loss.

Goode et al. (2015) conducted a systematic review of 27 exercise, diet, and/or weight management interventions for cancer survivors that were delivered via non-face-to-face methods (telephone, short-message service, print, and/or Internet). These researchers found that of those interventions that measured weight loss from pre- to post-treatment, effect sizes varied between 0.09 and 0.75, representing a 1.3–10.6% reduction in baseline weight. In comparison, the current study yielded results similar to or within this range, with effect sizes of 0.30 and 0.37 at post-treatment and 12-months follow-up, and 1.09 and 2.73% reduction in weight at post-treatment and 12-months follow-up.

Weight loss maintenance is very difficult to achieve, with an estimated 10–20% of formerly obese individuals being able to maintain significant weight loss long-term, and rapid weight gain being common (Uhley and Jen, 2007; Tylka et al., 2014). Researchers who have investigated the rapid-cycling of weight loss and regain (often referred to as “yo-yoing”) have found that such weight cycling is associated with insulin resistance (Lu et al., 1995), which may explain why some individuals experience great difficulty losing weight despite making changes in eating and activity that would otherwise be expected to generate weight loss. The study of weight-cycling in rats has found that such a pattern is associated with increased levels of 5-hydroxymethyl-2′-deoxyuridine, which is a marker of oxidized DNA damage and a risk factor of breast cancer (Djuric et al., 1996; Uhley and Jen, 2007). Thus, a history of repeated weight loss-gain cycling may place some women at increased risk of developing breast cancer (Uhley and Jen, 2007). Anecdotally, several of the women in the current sample described a longstanding history of repeated weight loss attempts via a number of various programs; further exploration of this theory may be warranted.

### Behavioral Health

Intuitive eating improved from pre- to post-intervention (*d* = 0.91) and this large effect was maintained at 12-months follow-up (*d* = 1.12). These results can be interpreted in comparison to other non-face-to-face lifestyle interventions for cancer survivors (Goode et al., 2015), which found that most (*n* = 9) of these sorts of interventions had a small effect on diet at post-treatment (*d* = 0.2–0.49), while four had a large effect (*d* ≥ 0.8), another four had a negligible effect, and two studies had a moderate effect (*d* = 0.5–0.79).

The HLM-ABC program demonstrated a medium effect (*d* = 0.72) on movement, with the sample increasing from an average level of “moderately active” at pre-treatment to a level of “active” at post-treatment. This effect, albeit small (*d* = 0.21), was maintained at 12-months follow-up. Goode et al. (2015) meta-analysis of non-face-to-face lifestyle interventions for BCSs concluded that when it comes to longer-term maintenance of PA and diet outcomes, effects are small (*d* < 0.49) or not reported at all. Therefore, it is a strength of the current study to have collected this follow-up data, and to have yielded a large sustained effect on eating habits; the small sustained effect on PA is consistent with other non-face-to-face lifestyle interventions for this population.

### Psychosocial Health

Participants’ level of motivation increased from pre-intervention to 6-months and 12-months follow-up. According to categorical analysis, the extent to which a person’s motivation changed from pre-intervention to 12-months follow-up also appeared to be associated with how much their weight changed during this time; those women whose motivation decreased during this time were more likely to have also gained weight, those who did not experience a change in motivation were also more likely to have not experienced a significant change in weight (<5% reduction in baseline weight), and those women whose motivation increased were more likely to have lost weight of a significant (>5% reduction in baseline weight) or insignificant (<5% reduction in baseline weight) amount.

The qualitative findings also converge to illustrate a positive change in motivation and general attitudes toward change. Participants described adopting an approach to change that is more gradual and continuous, and being more committed to prioritizing self-care (including through healthy eating and moving).

Contrary to expectations, exercise self-efficacy scores (E-SES) decreased between baseline and all subsequent time points. This quantitative result suggests that participants’ confidence in their ability to be in control of their exercise habits decreased slightly, which deviates from the qualitative themes regarding *feeling efficacious* (Table 3, item 2.1). Furthermore, a decrease in exercise self-efficacy seems to contradict the finding that self-reported levels of exercise (as measured by the GLTEQ) increased, on average. It is possible that self-efficacy may not have mediated or been directly related to actual levels of activity, which is a finding that has been reported by other studies of remotely delivered PA interventions for BCSs (Rabin et al., 2006; Vallance et al., 2007; Valle et al., 2015; Forbes et al., 2017).

Another possible explanation for this unexpected decrease in E-SES is that the HLM-ABC program was developed and implemented in close collaboration with two dieticians. Participants were provided guidelines regarding healthy eating and were given individualized feedback on their food diaries from one of the dieticians. However, the program did not include a similar degree of integration of PA guidelines or collaboration with an exercise or rehabilitation specialist. Therefore, as a result of the program’s relative emphasis on eating and dietetic influence, the women in the HLM-ABC program may have felt more confident in their abilities to persist in their eating goals than in their movement goals, which might explain the observed discrepancy between changes in nutrition versus exercise self-efficacy.

#### Psychological Well-Being

The current sample’s QoL did not change from pre-treatment to post-treatment, but decreased slightly between pre-treatment and 6-months follow-up, with a small effect. By 12-months follow-up, QoL had returned to a baseline level. A minimum change of 6 points on the FACT-B total score has been identified as clinically meaningful (Eton et al., 2004), and therefore, this temporary reduction (of 3.85 points) at 6-months is not likely to have been significant. This transient increase in distress may be explained by the fact that the intervention promoted increased self-awareness of (maladaptive) emotions and thoughts, and as described by a number of participants, unveiled longstanding and/or sensitive issues (e.g., feelings of low self-worth, neglect of one’s needs/values). Such insight was encouraged while simultaneously encouraging participants to refrain from engaging in familiar yet problematic coping behaviors (e.g., emotional eating, inactivity) and experiment with new, healthier strategies (e.g., increased self-care, greater movement, mindful eating), which may have understandably generated temporary feelings of discomfort or distress.

#### Self-Concept

Women in this sample were slightly less distressed about their bodies upon completing the HLM-ABC program and still 1 year beyond, compared to before starting the program. This increase in self-acceptance occurred in light of perceived physical change for some, and *despite the absence* of perceived physical change for others. This finding is consistent with evidence that engaging in PA for pleasure rather than for weight loss is associated with well-being and improved body image (Homan and Tylka, 2014). This outcome suggests that in the absence of quantifiable weight loss, BCSs can improve their self-concept and still achieve an overall sense of feeling healthier.

### Summary and Implications

The aim of this research was to evaluate the potential health impacts of the HLM-ABC program, across a number of biopsychosocial health indicators. Although participants in the current study achieved varying degrees of weight loss (and two gained weight), they also demonstrated improvements (varying from small to large magnitude) in other domains of health and well-being, including intuitive eating, PA level, motivation, nutrition self-efficacy, and BIS. In addition to these quantitative outcomes, the women reported themes of increased self-awareness, feelings of empowerment and self-efficacy, increased skillfulness in approaching their health goals, and enhanced self-acceptance. These mixed-method findings suggest that positive behavioral, emotional, and attitudinal health changes can occur even when physical outcomes (i.e., weight loss) are modest and in spite of very real, unmodifiable barriers to weight loss (hormonal treatments, metabolic/insulin resistance related to chronic dieting, genetics).

Mainstream research and health care tend to conceptualize health according to quantifiable outcomes (e.g., BMI, muscle mass, measures of body circumference, biomarkers), with weight often being the primary indicator. However, this medicalized approach to health has been criticized by proponents of a “weight-inclusive” (versus “weight-normative”) approach to health (Tylka et al., 2014). Among the problems associated with a weight-centric system are: weight-cycling and its related adverse health impacts (e.g., heightened risk of bone fractures and gallstones, muscle atrophy, hypertension, chronic inflammation; Rzehak et al., 2007; Nilsson, 2008; Tylka et al., 2014); failure to diagnose legitimate health conditions in individuals whose weight appears ‘normal; false diagnosis and prescription of weight loss interventions for individuals who are considered “overweight” but are otherwise healthy (Tylka et al., 2014); establishment of weight goals that are often unrealistic and generate chronic feelings of failure (Jeffery et al., 2000; Bacon et al., 2005; Wing and Phelan, 2005); absence of empirical evidence that a higher BMI actually *causes* poor health (Bruno, 2017); and harmful stigma presenting within oneself, between individuals and their health care providers, and amidst society at large. Furthermore, a weight-normative approach to health fails to account for the range of factors that can influence a woman’s body weight and shape that are beyond her control (e.g., genetics, age, hormonal status, metabolic functioning, disease- and/or treatment-related weight gain, surgical body changes) and can therefore impede her sense of autonomy and competence with respect to managing her own health—experiences that are considered fundamental to motivation and self-efficacy (Ryan and Deci, 2017).

The findings of this study suggest that women with a history of BC are capable of modifying one or various aspects of their well-being (e.g., improved body image, increased physical activity) and that such changes may or may not coincide with clinically significant weight loss. The findings from this study and developing sociocultural and academic perspectives around “weight inclusivity” invite us to reflexively and critically examine the validity of assigning body weight as the primary (best) measure to evaluate success/change when it comes to helping BCSs create meaningful and lasting improvements to their health. Future research in this area is warranted, including investigation of potential correlations between long-term health outcomes (e.g., cancer recurrence, mortality) with psychosocial and behavioral variables (e.g., body image, self-efficacy, intuitive eating habits) irrespective of weight loss.

### Study Limitations

A major limitation of this study is its small sample size. Although the sample represented a wide age range (29–71 years), time elapsed since treatment (ranging from 6 to 64 months) and annual income, the majority self-identified as White/Caucasian (nearly 79%) and as having a university-level education (64%). Results cannot be assumed to reflect the experiences of all BCSs who are seeking to make changes to their weight and/or lifestyle, especially those with various cultural and educational backgrounds.

Although an exclusion criterion for this research was participation in other types of health-promoting programs during their involvement in the study, actual data about this, or other potentially confounding variables (e.g., changes in health/illness), that could have influenced the results was not collected.

Furthermore, the small sample data were not amenable to statistical analyses that carry assumptions of normality and adequate power, thus were limited to exploratory analyses and reporting of effect sizes to estimate the magnitude of the HLM-ABC intervention’s potential. Notwithstanding these limits, a small sample size was appropriate given the aim of this study, which was to pilot the feasibility and acceptability of a novel intervention and estimate effect sizes, power, and sample size for later-phase trials.

## Conclusion

This study evaluated the HLM-ABC program’s preliminary effectiveness through triangulation of various physical, behavioral, and psychosocial outcome data to determine whether the intervention was successful in supporting sustained (i.e., 1-year follow-up) improvements in a number of health indicators rather than relying exclusively or unduly on weight. Results demonstrated quantitative and qualitative trends of sustained but modest weight loss, increased intuitive eating, higher levels of PA, increased motivation and self-efficacy (regarding eating habits), and improved body image.

The preliminary effectiveness of the HLM-ABC pilot program is quite promising in light of the fact that it was a relatively self-directive, at-home intervention that did not impose restrictions or strict prescriptions. In other words, the HLM-ABC program did not so much instruct participants on *what* to do to establish a healthy lifestyle, but rather taught principles and practices for how to develop healthier lifestyle habits that suit each woman’s unique situation, needs and preferences. Furthermore, considering that this online intervention was implemented entirely through text-based interaction (i.e., no in-person or video meetings), it is interesting to think how much greater the impacts of the program could be in the future with the incorporation of additional opportunities for human connection, accountability and external structure especially in the current context where video-conferencing is ubiquitous. Additionally, future implementations of the program could be improved by incorporating greater guidelines (i.e., 30 min per day of moderate to vigorous daily activity) and direct clinical input from collaborators with a background in exercise/physiology to compliment the relative degree of eating guidelines and involvement of dieticians in this intervention. Overall, the results herein suggest that the HLM-ABC program, and holistic person-centered interventions more broadly, have potential to help BCSs adopt and maintain a healthier lifestyle following treatment.

## Data Availability Statement

The original contributions presented in the study are included in the article/Supplementary Material, further inquiries can be directed to the corresponding author/s.

## Ethics Statement

The studies involving human participants were reviewed and approved by Sunnybrook Health Sciences Centre (SHSC)’s Research Ethics Office (339-2014) and York University’s Office of Research Ethics (Sunnybrook Approval –339-2014). The patients/participants provided their written informed consent to participate in this study.

## Author Contributions

DM, KF, and SY contributed to conception and design of the study, recruitment, intervention implementation and data collection. DM organized the database and conducted analyses in collaboration with KF. DM wrote the first draft of the manuscript. KF contributed written sections. All the authors contributed to manuscript revision, read, and approved the submitted version.

## Conflict of Interest

The authors declare that the research was conducted in the absence of any commercial or financial relationships that could be construed as a potential conflict of interest. The handling editor MF declared a shared affiliation with the author KF at the time of review.

## Publisher’s Note

All claims expressed in this article are solely those of the authors and do not necessarily represent those of their affiliated organizations, or those of the publisher, the editors and the reviewers. Any product that may be evaluated in this article, or claim that may be made by its manufacturer, is not guaranteed or endorsed by the publisher.
