# Supplementary material for: ‘Weighing’ Losses and Gains: Evaluation of the Healthy Lifestyle Modification After Breast Cancer Pilot Program
Source: Front Psychol. 2022 Mar 25;13:814671. doi: 10.3389/fpsyg.2022.814671 (PMC8992775; doi:10.3389/fpsyg.2022.814671)
Supplement: Supplementary file 1 [file Data_Sheet_1.docx]

# Post-Treatment Semi-Structured Interview Questions

**Please share with me your experience of the *Healthy Lifestyle Modification after Breast Cancer* program overall**

- - What was it like to take part in this program?
  - In what ways was the program helpful to you? How so?
- What did you like most about the program? What did you like least?
- If you recall, the program covered a range of topics, including… (refer to list). Which session or topic did you like most? Please elaborate.
  - Which session or topic did you like the least? Please elaborate.
- What did you think about the length of the program? Did you find 10 topics to be too much, not enough, or just the right amount?
- Was there anything you felt was missing from the program or that you would have hoped to focus on more?
- How did you find the homework assignments? (Regardless of whether they *liked* doing them or not) Were these a useful part of the program?
  - What about the amount of homework? Did you find it to be too much, not enough, or just the right amount?
- How did you find using the diaries to track your behaviours (food and activity)? Were these a useful part of the program?
  - What were your expectations going into the program? (probe re: whether these were met).
- Did the program fall short of your expectations in any way?
  - What was your experience of having this program offered as part of a group with other women, as opposed to doing this one-on-one with a facilitator online? Did you find the social aspect to be a helpful feature of the program? Did you feel supported by or connected with your fellow group members?
  - What, if any, were the challenges to your participation?

**Please share with me your experience of the online format of the program**

- - What was it like to take part in an ***online*** (versus in-person) program?
  - Did you find the Moodle website user-friendly? What did you like/dislike about it?
  - How did you find the weekly videos? Was this a useful way of delivering information online?
  - How did you find using the discussion board? Was it a valuable aspect of the program?
  - Do you have any suggestions about how to improve the online delivery of this program?

**Please share with me your experience with your group facilitators**

- How did you find the involvement of your group facilitators?
- What other support from the facilitators do you think would be beneficial in the future?

**I would now like to ask you some questions to learn about whether or how your behaviours and views might have changed since participating in this program.**

- Do you feel that you are living a healthier lifestyle now, after having participated in the healthy lifestyle group? Please explain.
  - If so, what occurred throughout your participation that has led to this progress?
  - If not, why do you feel that your participation did not lead to significant changes?
- Since participating in this program, how, if at all, have your eating habits changed?
- Since participating in this program, how, if at all, have your physical activity habits changed?
  - How confident are you that you can maintain the changes that you have made? How do you plan on doing so?
- What would you say the biggest challengers or barriers might be for you going forward in maintaining a healthy weight or lifestyle in general?
- Regardless of whether or not you have lost weight, how do you currently feel about your weight/body? Has this changed at all after having participated in the lifestyle group?

**Please share with me any other thoughts**

- Is there anything else you would like to share with us about your experience in this program?
